# Genome Sequences of Three *Apple chlorotic leaf spot virus* Isolates from Hawthorns in China

**DOI:** 10.1371/journal.pone.0161099

**Published:** 2016-08-12

**Authors:** Wei Guo, Wenyan Zheng, Mei Wang, Xiaohong Li, Yue Ma, Hongyan Dai

**Affiliations:** 1 College of Horticulture, Shenyang Agricultural University, 120 Dongling Road, Shenyang, Liaoning, 110866, China; 2 College of Agronomy, Eastern Liaoning University, 325 Wenhua Road, Dandong, Liaoning, 118003, China; Wuhan Botanical Garden, CHINA

## Abstract

The genome sequences of *Apple chlorotic leaf spot virus* (ACLSV) isolates from three accessions of hawthorns (*Crataegus pinnatifida*) grown at Shenyang Agricultural University were determined using Illumina RNA-seq. To confirm the assembly data from the *de novo* sequencing, two ACLSV genomic sequences (SY01 and SY02) were sequenced using the Sanger method. The SY01 and SY02 sequences obtained with the Sanger method showed 99.5% and 99.7% nucleotide identity with the transcriptome data, respectively. The genome sequences of the hawthorn isolates SY01, SY02 and SY03 (GenBank accession nos. KM207212, KU870524 and KU870525, respectively) consisted of 7,543, 7,561 and 7,545 nucleotides, respectively, excluding poly-adenylated tails. Sequence analysis revealed that these hawthorn isolates shared an overall nucleotide identity of 82.8–92.1% and showed the highest identity of 90.3% for isolate YH (GenBank accession no. KC935955) from pear and the lowest identity of 67.7% for isolate TaTao5 (GenBank accession no. EU223295) from peach. Hawthorn isolate sequences were similar to those of ‘B6 type’ ACLSV. The relationship between ACLSV isolates largely depends upon the host species. This represents the first comparative study of the genome sequences of ACLSV isolates from hawthorns.

## Introduction

*Apple chlorotic leaf spot virus* (ACLSV), a representative species of genus *Trichovirus* in family Betaflexiviridae [1], is distributed worldwide and can infect most fruit tree species of family Rosaceae, including apple, pear, peach, plum, almond, apricot, cherry and hawthorn [2]. ACLSV is a latent virus that usually cannot cause obvious symptoms in cultivars of apples and pears. The severity of the symptoms caused by ACLSV shows a strong association with plant species and virus strains [2–3]. The virus can cause severe symptoms in many pomes and stone fruit trees, including plant dysplasia and less robust plant growth. The main disease agent of apples and pears grafted onto susceptible rootstocks can be attributed to co-infection of ACLSV with *Apple stem grooving virus* and/or *Apple stem pitting virus* [4]. ACLSV is mainly spread through the grafting, pruning, or propagation of materials and nematodes, and has not yet been found to be transmitted through seeds or natural media. Because of the inadequate development of virus-free plantlets in recent years, virus transmission caused by grafting now represents a major threat to the fruit industry.

ACLSV, which is 640–760 nm in length, is a positive-sense, single-stranded RNA particle. The ACLSV genome is composed of 7474–7561 nucleotides, excluding the poly-adenylated tail, with untranslated regions of 150 and 215 nucleotides at its 5ʹ- and 3ʹ-termini, respectively [5]. The complete nucleotide sequence contains three overlapping open reading frames (ORFs) that encode a 216-kDa replication-associated protein (Rep), a 50-kDa movement protein (MP), and a 22-kDa coat protein (CP) [6–7]. The CP is the only constitutive protein, and it has a relatively conserved gene sequence.

Although GenBank includes many partial or complete genome sequences of ACLSV, no sequence of an ACLSV isolate from a hawthorn was available prior to our present study. Some studies have indicated that ACLSV has many variants with different serological reactivity and strains that reflect different host species and geographical distributions [8–9].

To better understand the molecular characteristics of ACLSV isolates from hawthorns, the genome sequences of these ACLSV isolates were determined, and the nucleotide and amino acid identities and phylogenies were analyzed.

## Materials and Methods

### Plant materials

Young plant leaves and fruits of the *Crataegus pinnatifida* accessions used in this study were collected from Shenyang Agricultural University.

### RNA extraction

Total RNA was extracted from 100 mg of hawthorn leaves using a modified CTAB method [10].

### High-throughput sequencing

All cDNA library preparation and sequencing reactions were carried out by the Biomarker Technology Company. Paired-end library preparation and sequencing were performed following standard Illumina methods using a DNA sample kit. The cDNA libraries were sequenced on the following Illumina sequencing platforms: HiSeq^TM^ 2000 for SY01 and HiSeq^TM^ 2500 for SY02 and SY03.

### Primer design and reverse transcription-polymerase chain reactions

Primers for the amplification of genome fragments from SY01 and SY02 (S1 Table and S2 Table) were designed based on transcriptome data and synthesized by GENEWIZ, Inc. (Beijing, China). Reverse transcription reactions were performed at 37°C for 30 min with PrimeScript^®^ RT reagent kit (TaKaRa, Dalian, China) according to the manufacturer’s instructions. PCR reactions were carried out in 20 μL total volumes with reaction mixtures that contained 1 μL cDNA, 1.6 μL of each dNTP (2.5 mM), 2.0 μL 10× PCR buffer, 1.0 μL MgCl_2_ (25 mM), 0.5 μL of each primer (10 μM), 0.2 μL Taq DNA polymerase (Promega, Shanghai, China) and ddH_2_O to yield a 20 μL final volume.

### Cloning, sequencing, sequence assembly and analysis

PCR products were gel-extracted and ligated into a pMD18-T vector (TaKaRa, Dalian, China). Positive clones for each product were sequenced at Beijing Genomics Institute, China.

**Fig 1:**
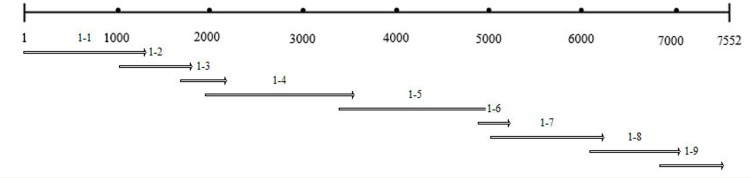
An amplification diagram for the full-length sequence of SY01. A total of nine fragments, 1–1 to 1–9, were used to assemble the 7.5 kb gene sequence for SY01. The overlapping segments were between 100 and 200 bp.

The complete genome sequences of ACLSV from hawthorns were assembled with overlapping fragments of more than 100 bp, as shown in the diagram with SY01 as a representative example (Fig 1). Nucleotide and amino acid identities were compared using the DNAMAN software package (Version 5.2.2.0). Sequences of other ACLSV isolates were downloaded from the National Center for Biotechnology Information (NCBI), including MO-5 (accession no. AB326225), B6 (accession no. AB326224), A4 (accession no. AB326223), P-205 (accession no. D14996), RC (accession no. HE980332), QD-13 (accession no. KJ522693), JB (accession no. KC935956), KMS (accession no. KC935954), YH (accession no. KC935955), P863 (accession no. M58152), PBM1 (accession no. AJ243438), Z1 (accession no. JN634760), Z3 (accession no. JN634761), TaTao5 (accession no. EU223295) and Bal1 (accession no. X99752). A multiple sequence alignment was performed using Clustal X (http://www.clustal.org) [11]. Phylogenetic trees were generated using the multiple sequence alignment results and constructed by the neighbor-joining method with 1000 bootstrap replicates with MEGA software (version 6.0). Data about the ACLSV isolates used for sequence analysis and alignment are listed in Table 1.

**Table 1:** Origin and GenBank accession numbers of ACLSV isolates used for sequence alignment and phylogenetic analysis.

Isolate	Host	Country	GenBank No.	Reference
SY01	Hawthorn	China	KM207212	This study
SY02	Hawthorn	China	KU870524	This study
SY03	Hawthorn	China	KU870525	This study
MO-5	Apple	Japan	AB326225	[12]
B6	Apple	Japan	AB326224	[12]
A4	Apple	Japan	AB326223	[12]
P-205	Apple	Japan	D14996	[6]
RC	Apple	India	HE980332	[13]
QD-13	Apple	China	KJ522693	[3]
JB	Pear	China	KC935956	[14]
KMS	Pear	China	KC935954	[14]
YH	Pear	China	KC935955	[14]
P863	Plum	France	M58152	[15]
PBM1	Plum	Germany	AJ243438	[16]
Z1	Peach	China	JN634760	[7]
Z3	Peach	China	JN634761	[7]
TaTao5	Peach	America	EU223295	[17]
Bal1	Cherry	France	X99752	[18]

## Results

### Assembly of the hawthorn ACLSV genome sequence

**Fig 2:**
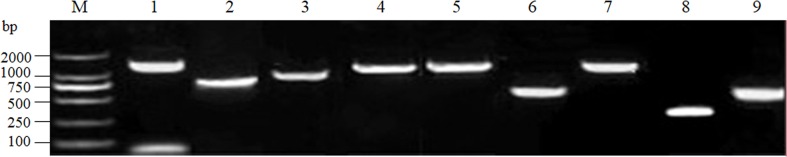
The amplification of each fragment of SY01 by RT-PCR. M represents the DL2000 marker, 1–9 represent the nine fragments of SY01.

The SY01, SY02 and SY03 sequences of ACLSV from three accessions of hawthorns, with 7,543, 7,561 and 7,545 nucleotides, respectively, were first determined using Illumina RNA-seq. Then, SY01 and SY02 were assembled by RT-PCR to confirm the transcriptome data. The sizes of nine specific fragments of SY01 obtained by RT-PCR were as follows: 1,652, 799, 987, 1,222, 1,235, 699, 1,603, 356 and 514 bp (Fig 2). For SY02, the sizes of the nine amplification fragments were as follows: 1,146, 766, 797, 974, 983, 1,015, 1,207, 1,162 and 775 bp. The reassembled full-length sequences of SY01 and SY02 showed 99.5% and 99.7% nucleotide identities, respectively, with the transcriptome data, which confirmed the reliability of our transcriptome data.

### Genomic characterization and sequence analysis

The three complete nucleotide sequences of ACLSV from hawthorns, SY01, SY02 and SY03, consisted of 7,543, 7,561 and 7,545 nucleotides, respectively, excluding the poly-adenylated tail. We made these sequences available in GenBank with the accession numbers KM207212, KU870524 and KU870525, respectively. The genome of the SY02 isolate was longer than the other two isolates because of differences in nucleotide numbers in the 5ʹ-untranslated regions (5ʹ-UTR), as shown in Table 2. Sequence analysis showed that these three hawthorn isolates shared an overall nucleotide identity of 82.8–92.1%. The complete nucleotide sequence of these three hawthorn isolates contained three overlapping ORFs that were 5,634, 1,383 and 582 nucleotides in length (ORF 1, 2, and 3, respectively). All three isolates had the same number of nucleotides and amino acids. ORFs of the MO-5 isolate from apples consisted of the same number of nucleotides as those from hawthorn isolates.

**Table 2:** Sequence comparison of the complete genome and different genomic regions between ACLSV SY01 and isolates SY02, SY03 and fifteen ACLSV isolates reported previously.

Host	Isolate	Genome		5’ UTR		Rep ^a^			MP ^a^			CP ^a^			3’ UTR	
		nt^b^	nt%^c^	nt	nt%	nt	nt%	aa%^c^	nt	nt%	aa%	nt	nt%	aa%	nt	nt%
Hawthorn	SY01	7,543	-	138	-	5,634	-	-	1,383	-	-	582	-	-	212	-
	SY02	7,561	92.1	157	85.4	5,634	91.8	95.3	1,383	94.0	96.1	582	95.9	98.5	211	96.2
	SY03	7,545	83.1	139	93.5	5,634	81.8	91.4	1,383	86.7	90.7	582	90.9	97.4	213	85.6
Apple	MO-5	7,561	82.1	152	84.9	5,634	80.6	90.0	1,383	85.3	96.1	582	90.4	94.8	216	83.3
	B6	7,553	75.7	151	68.0	5,649	73.8	80.9	1,377	79.7	93.5	582	84.5	96.9	194	80.2
	A4	7,548	75.0	151	70.6	5,658	73.7	82	1,377	81.0	93.3	582	85.9	96.4	180	73.1
	P-205	7,552	76.0	151	68.6	5,658	74.1	82.1	1,374	80.0	92.4	582	84.9	90.7	187	74.1
	RC	7,525	74.1	150	64.9	5,649	72.8	78.7	1,356	79.5	80.7	582	84.4	93.8	167	63.4
	QD-13	7,557	75.5	151	72.6	5,652	73.8	81.1	1,377	81.9	82.6	582	85.1	93.8	195	77.4
Pear	JB	7,560	82.9	151	86.8	5,637	81.4	89.1	1,377	85.3	98.0	582	90.2	97.9	213	88.8
	KMS	7,528	82.7	119	79.7	5,637	81.3	90.0	1,377	87.9	90.4	582	90.9	96.9	213	87.0
	YH	7,528	83.0	119	79.7	5,637	81.7	89.9	1,377	87.3	90.9	582	91.4	96.4	216	88.8
Plum	P863	7,555	74.7	151	68.6	5,655	73.4	82.0	1,383	77.5	93.5	582	82.5	94.8	190	68.6
	PBM1	7,545	75.7	151	70.1	5,652	74.0	81.7	1,383	79.8	93.9	582	84.4	91.7	183	75.9
Peach	Z1	7,552	74.6	151	69.9	5,652	73.4	81.6	1,383	78.7	92.8	582	82.8	95.3	190	76.1
	Z3	7,552	74.6	151	68.6	5,652	73.4	81.7	1,383	78.6	92.8	582	84.2	95.3	190	75.1
	TaTao5	7,474	68.1	159	49.1	5,643	69.3	74.2	1,341	65.9	84.8	582	70.3	74.1	143	46.3
Cherry	Bal1	7,549	74.8	148	61.2	5,664	74.0	80.7	1,383	78.0	92.2	582	82.7	88.6	178	60.6

^a^ Rep, replication-associated protein; MP, movement protein; CP, coat protein.

^b^ nt, nucleotide; aa, amino acid.

^c^ nt%, aa%, the nucleotide and amino acid identities between SY01 and other isolates.

Sequence analysis revealed that SY01 and SY02 had high similarity, with 92.1% nucleotide identity and 95.3%, 96.1% and 98.2% amino acid sequence identity based on a comparison of the three ORFs. SY03 shared only 82.8–83.1% nucleotide identity and 90.7–97.4% amino acid similarity with SY01 and SY02. The nucleotide sequence and amino acid identities of the whole genome as well as different genomic regions between hawthorn isolates and fifteen previously reported ACLSV isolates were analyzed. Table 2 shows a sequence comparison between SY01 and other isolates. The three hawthorn isolates showed the highest nucleotide identity with YH (90.3%) and the lowest with TaTao5 (67.7%). The amino acid identities between SY01 and the three isolates from pear (JB, KMS and YH) were all greater than 89%, showed very high homology, and always clustered together according to our phylogenetic analysis. Rep, MP and CP of ACLSV isolates from hawthorns shared 74.2–94.1%, 58.3–95.0% and 74.1–99.0% overall amino acid identities, respectively, compared with those of other isolates shown in Table 2.

### Variability of the CP gene among ACLSV isolates

**Fig 3:**
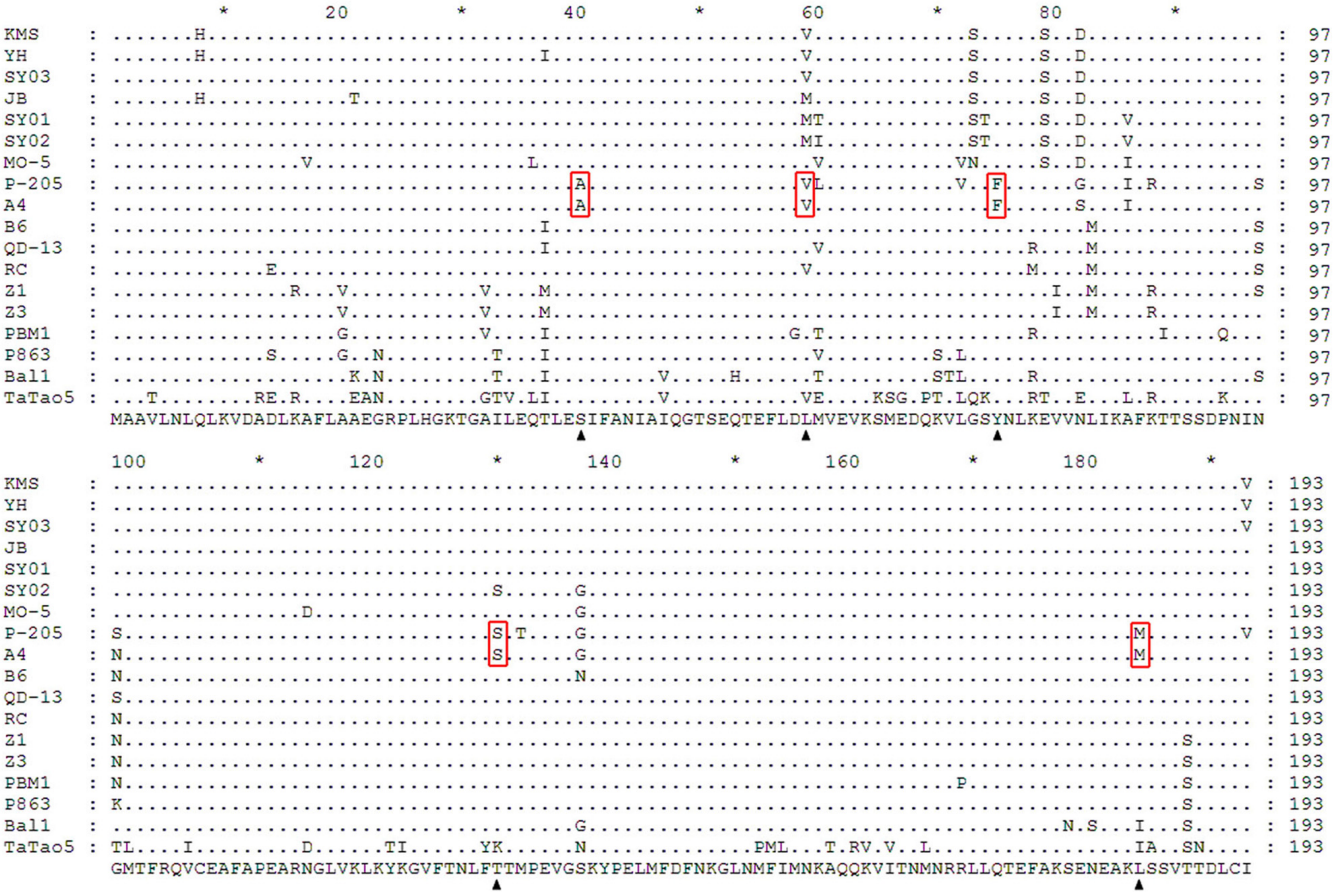
Multiple sequence alignments based on coat protein amino acid sequences of ACLSV isolates reported previously. Arrows indicate the 5 characteristic positions among ‘B6 type’ ACLSV isolates. Conserved amino acids of the ‘P-205 type’ are marked with red boxes. Consensus amino acid sequences are shown in the bottom line, and diverse amino acids are shown in the comparison.

The CP was most conserved, having more than 90% amino acid identity between SY01 and other isolates, except for two distinct isolates (Ball and TaTao5). All CPs of the eighteen isolates corresponded to 582 nucleotide genes that encoded 193 amino acids. A multiple alignment based on the eighteen amino acid sequences of ACLSV CP also illustrated the sequence conservation of the CP gene, especially from amino acid sites 100–193 (Fig 3). The ‘B6 type’ (S40-L59-Y75-T130-L184) and ‘P-205 type’ (A40-V59-F75-S130-M184) of ACLSV are classified by the five characteristic sites of the CP, which have been frequently described in previous studies [12,19]. The three hawthorn isolates were each similar to the ‘B6 type’. All ACLSV isolates discussed in this present study belonged to the ‘B6 type’, except for the A4 isolate. However, compared with the B6 isolate, SY01 and SY02 had amino acid M at position 59, which was consistent with the JB isolate from pears; SY03 and the other two pear isolates had amino acid V in that same position, and SY01 had a different amino acid, S, at position 130. In an alignment of the reported CP amino acid sequences of ACLSV isolates, the TaTao5 isolate had the fewest conserved amino acids. The amino acid motif (S40-V59-Y75-K130-I184) of the TaTao5 isolate only had two conserved amino acids with B6 at sites 40 and 75.

### Phylogenetic analysis

**Fig 4:**
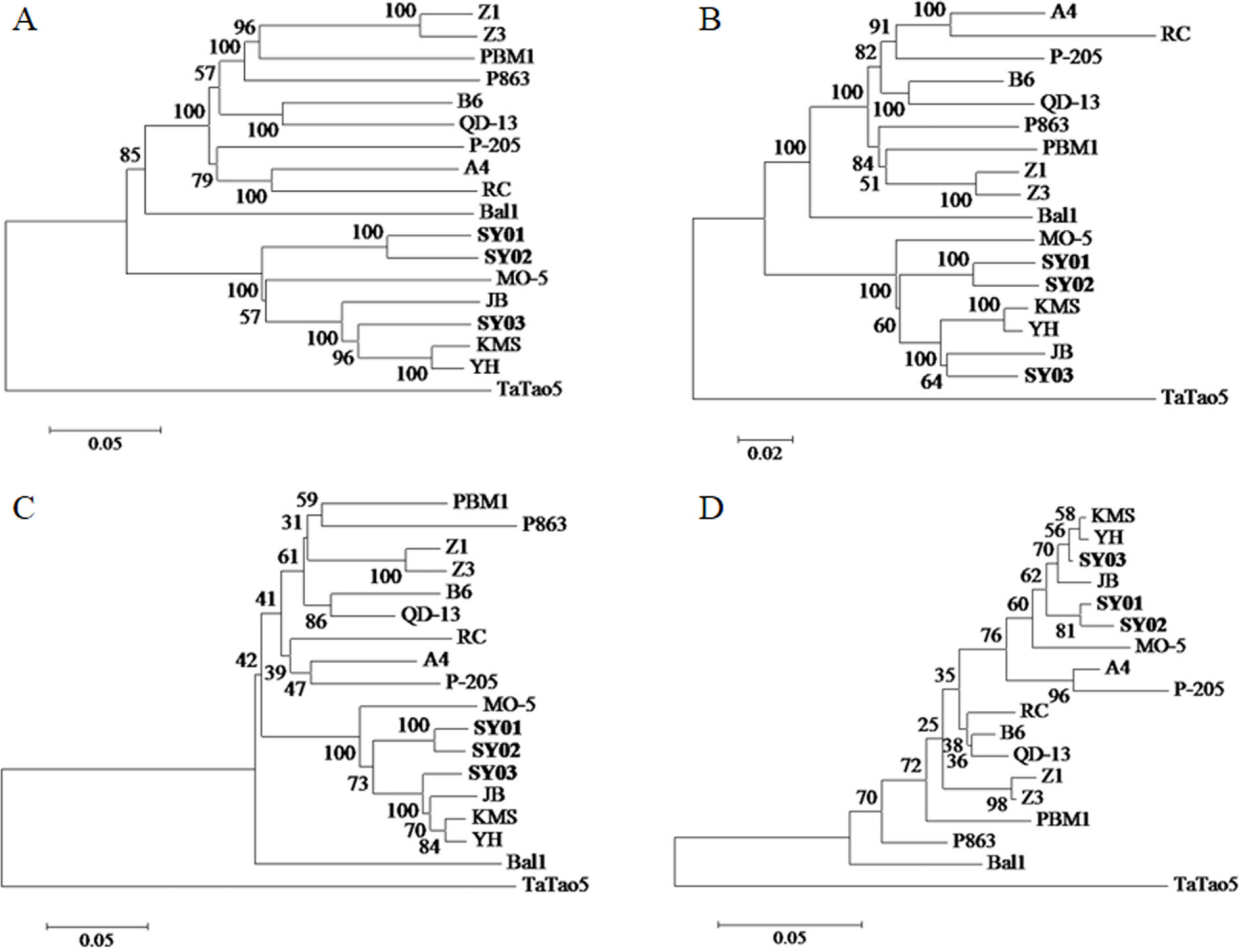
**The phylogenetic trees of ACLSV isolates were based on sequences of the whole genome (A), replication-associated proteins (B), movement proteins (C), and coat proteins (D)**.

According to our phylogenetic analysis based on nucleotide and amino acid sequences of eighteen ACLSV isolates, similarities of the ACLSV isolates showed a strong association with their respective host species. Our analysis of the phylogenetic tree generated from the whole genome (Fig 4A) revealed that these isolates could be mainly divided into four distinct clades. The peach isolate TaTao5 and the only cherry isolate Bal1 both individually formed separate clades. The apple, pear and hawthorn isolates belonged to pome fruit trees, and those isolates from pear and hawthorn along with apple isolate MO-5 formed another clade. Finally, isolates from stone fruit trees, including Z1 and Z3 from peach and PBM1 and P863 from plum, were grouped into the last clade along with five other apple isolates. This grouping also applied to the phylogenetic trees for Rep (Fig 4B) and MP (Fig 4C). Many sub-clades are always present in trees. SY03 belonged to the same sub-clade with the three pear isolates, while the isolates from peach (except TaTao5) and plum formed another sub-clade.

## Discussion

Previously, viral RNA was extracted from purified virus [6] and first- and second-strand cDNA were obtained by generating cDNA libraries [20]. Recently, next-generation sequencing (NGS) has been developed and has been applied to allow for rapid diagnosis and detection. Both emerging and known plant viruses can be easily discovered by high-throughput sequencing [21]. Liu et al. [22] determined the whole genome sequence of a Chinese isolate of *Pepper vein yellows virus* using deep sequencing of small RNAs. Bejerman et al. [23] discovered a new enamovirus and obtained genome sequences from alfalfa plants that showed dwarfism symptoms by *de novo* sequencing, which was confirmed by the Sanger method. In this present study, three ACLSV isolates from hawthorns were determined by Illumina RNA-seq, and two of them were validated by RT-PCR, which showed a high degree of similarity with the transcriptome data. The study of Khalifa et al. [24] also mentioned that the *de novo* assembled genomes from Illumina were 99.3–100% similar to Sanger sequencing results. Together, these findings established the veracity and reliability of sequence data from NGS.

Through a comparison of hawthorn isolates and fifteen reported isolates, we found that ORF3 was the most conserved, while ORF1, ORF2 and the 3ʹ-UTR and 5ʹ-UTR were relatively diverse, especially ORF1, which had a highly variable region [18]. ORF1 of ACLSV encodes Rep, including methyltransferase, protease, helicase, and RNA-dependent RNA polymerase. The poorly conserved region was mapped to the protease. Zhu et al. [14] have suggested that the sequence of the hypervariable region might be related to the phylogenetic evolution of the virus. The CP at the C-terminus of the plant virus was relatively conserved. We can conclude that most of the variability was present in the N-terminal domain of the CP, which overlapped with the C-terminus of MP, whereas the C-terminus of CP was significantly less variable [8], as shown in Fig 3. Conservation of CP corresponds with conservation of the entire genome sequence. Isolate TaTao5 had the fewest conserved amino acids, and had a distant relationship with the other isolates.

A comparison of the amino acid sequences of the CP revealed that only the A4 isolate showed the same amino acid combination (A40-V59-F75-S130-M184) as P-205 (Fig 3). Based on the phylogenetic trees, A4 was always grouped into the same subclade as P-205. This present study also confirmed that the classifications of the ‘B6 type’ and ‘P-205 type’ were reasonable. Yaegashi et al. [12] proposed that the specific combination of amino acid sites 40 and 75 (S40-Y75 or A40-F75) had a strong influence on viral accumulation and replication. From the diagram shown in Fig 3, it was evident that these two sites were highly conserved. Chen et al. [25] proposed the four phylogenetic types based on the three signature sites of the CP of ACLSV. From Chen’s classification standard, we can conclude that isolates from hawthorns and pears and MO-5 belong to group II (S40-Y75-S79), P-205 and A4 belong to group III (A40-F75-E79), and TaTao5 belongs to group IV (S40-Y75-T79), while the others belong to group I (S40-Y75-E79). There were many types of ACLSV CP, and virus variation is widespread in nature; however, the mutation mechanism has remained unclear to date.

Genome sequences of the presently reported eighteen ACLSV isolates were from Asia, Europe and America, and the respective hosts were Rosaceae fruit trees, including apple, pear, peach, plum, cherry and hawthorn. The eighteen isolates could be divided into four groups according to the phylogenetic analysis for trees generated based on whole genome sequences; this grouping was consistent with that of a previous study [3]. Among these groups, the apple isolates could be divided into two groups—MO-5 formed one group, while the other isolates formed another group. Niu et al. [7] proposed that two types of ACLSV isolates exist in peaches, the Z1 type and the TaTao5 type. Hawthorn isolates also formed two branches. We can conclude that isolates from the same fruit trees fall into the same or adjacent clades according to the phylogenetic clades. Currently, there is not sufficient evidence to show whether there is a correlation between different isolates and the original country of the host. Characterizing the molecular characteristics of ACLSV and the relationship between isolates and host species and origins will require further study to obtain new insights into virus population structure and evolution.

In summary, this study represents a comparative analysis of the whole genome sequences of ACLSV isolates from hawthorns and assessed sequence similarities and phylogenies among the eighteen ACLSV isolates that had been previously reported. Our present findings demonstrate that isolates from hawthorns and pears show a very close relationship, and the sequence identities of ACLSV isolates depend largely on the host species. This study also supports the notion that the classification of ‘B6 type’ and ‘P-205 type’ that had been reported [12] was reasonable and describes the variation in the CP of ACLSV. These findings may provide a basis for strain partitioning of ACLSV, which could lay a foundation for viral prevention and control.

## Supporting Information

S1 Table Primer sequences for the amplification of ACLSV isolate SY01. (DOC)

S2 Table Primer sequences for the amplification of ACLSV isolate SY02. (DOC)
