# Supplementary material for: Genome Sequences of Three Apple chlorotic leaf spot virus Isolates from Hawthorns in China
Source: PLoS One. 2016 Aug 12;11(8):e0161099. doi: 10.1371/journal.pone.0161099 (PMC4982687; doi:10.1371/journal.pone.0161099)
Supplement: S1 Table — (DOC) [file pone.0161099.s001.doc]

**S1 Table.** Primer sequences for the amplification of ACLSV isolate SY01.

| Primer | Nucleotide sequence  (5'-3') | Location  (nt) | Annealing temperature  (℃ ) | Product size  (bp) |
| --- | --- | --- | --- | --- |
| ACLSV1-1F | GTGTACACTCATATCGTGAGTAGAC | 1-25 | 55 | 1319 |
| ACLSV1-1R | GTGAAATCATCAGCCAGACC | 1300-1319 |  |  |
| ACLSV1-2F | GTGCCAATTCAAGATTTTCCA | 1300-1053 | 52 | 799 |
| ACLSV1-2R | TTTTCACTTTTGCTTTCTTGATG | 1809-1831 |  |  |
| ACLSV1-3F | GTTTAAATCAAACTGCCTGAGC | 1686-1707 | 52 | 514 |
| ACLSV1-3R | CCCAATCTGTTTCTCCAAATAA | 2178-2199 |  |  |
| ACLSV1-4F | GATAAGGTCGTCCAGAATGTGC | 1966-1987 | 55 | 1603 |
| ACLSV1-4R | CCTTGAGAACCCCTTCCATA | 3549-3568 |  |  |
| ACLSV1-5F | GATTGGAGTGAAAAGGGTG | 3400-3418 | 51 | 1652 |
| ACLSV1-5R | ACATCAAAAGCGGTGTAATC | 5032-5051 |  |  |
| ACLSV1-6F | GAATTTAGTCCCTGGTGCAG | 4888-4907 | 55 | 356 |
| ACLSV1-6R | GTGAACACCATGTTCGCCAA | 5224-5243 |  |  |
| ACLSV1-7F | GATTACACCGCTTTTGATGTG | 5032-5052 | 54 | 1222 |
| ACLSV1-7R | GAAGCTGTCAAAGAATCTGCAC | 6232-6253 |  |  |
| ACLSV1-8F | TCAGCCACGTTGGTGACTTC | 6089-6108 | 55 | 987 |
| ACLSV1-8R | GCAAAAGCCTCACAAACCTG | 7056-7075 |  |  |
| ACLSV1-9F | AATTCTGGAACAGACACTGG | 6845-6864 | 52 | 699 |
| ACLSV1-9R | GTAGTAAAATATTTAAAAGTCTACAGGC | 7516-7543 |  |  |
