# Supplementary material for: Genome Sequences of Three Apple chlorotic leaf spot virus Isolates from Hawthorns in China
Source: PLoS One. 2016 Aug 12;11(8):e0161099. doi: 10.1371/journal.pone.0161099 (PMC4982687; doi:10.1371/journal.pone.0161099)
Supplement: S2 Table — (DOC) [file pone.0161099.s002.doc]

**S2 Table.** Primer sequences for the amplification of ACLSV isolate SY02.

| Primer | Nucleotide sequence  (5'-3') | Location  (nt) | Annealing temperature  (℃) | Product size  (bp) |
| --- | --- | --- | --- | --- |
| ACLSV2-1F | CGCCGGGATACTGAAGAAGT | 1-20 | 54 | 1146 |
| ACLSV2-1R | TTGGCAACAGCAGATTGAACAT | 1125-1146 |  |  |
| ACLSV2-2F | GAGGAAGTTAGAAGGTTTGGAC | 983-1004 | 55 | 766 |
| ACLSV2-2R | TCACATCAGTAGCAGCACAT | 1729-1748 |  |  |
| ACLSV2-3F | ACTTGAACCCTCATACCCC | 1558-1576 | 53 | 797 |
| ACLSV2-3R | CATTGATTATCGCCTGCTT | 2336-2354 |  |  |
| ACLSV2-4F | CTGTGGGCAATGACTTTCTT | 2178-2197 | 56 | 974 |
| ACLSV2-4R | TGAGTTGTGCGACACTATCC | 3132-3151 |  |  |
| ACLSV2-5F | CCACGATAGACTCAAGGAG | 3025-3034 | 53 | 983 |
| ACLSV2-5R | AATATGGGCATCAGAACAC | 3989-4007 |  |  |
| ACLSV2-6F | CTTGGTCGCCAGTATGATTG | 3866-3885 | 58 | 1015 |
| ACLSV2-6R | CGAGTGTTTGCCCAGCCTTA | 4862-4881 |  |  |
| ACLSV2-7F | GCAGACAGGAGTTTGAAGA | 4707—4725 | 55 | 1207 |
| ACLSV2-7R | GGGTATGACTGGGATTGATG | 5894-5913 |  |  |
| ACLSV2-8F | GGGTCACAAATTGAAGATCG | 5721-5740 | 56 | 1162 |
| ACLSV2-8R | AGTGTCTGTTCCAGGATTGC | 6863-6882 |  |  |
| ACLSV2-9F | AGACAAAATCAGGCGAAGG | 6748-6766 | 53 | 775 |
| ACLSV2-9R | GTCTAAACACTCCAATTTAATACCA | 7498-7522 |  |  |
